# Ultrafast power Doppler imaging for RV myocardial perfusion assessment: histopathologic correlation and *in vivo* comparison—a pilot study

**DOI:** 10.1093/ehjimp/qyag035

**Published:** 2026-02-26

**Authors:** Naiyuan Zhang, Jerome Baranger, Mei Sun, Matteo Ponzoni, Nikan Fakhari, Maelys Venet, Luc Mertens, Jason Maynes, John G Coles, Mark K Friedberg, Olivier Villemain

**Affiliations:** Division of Cardiology, Labatt Family Heart Centre, Hospital for Sick Children, Toronto, Ontario, Canada; Division of Cardiology, Labatt Family Heart Centre, Hospital for Sick Children, Toronto, Ontario, Canada; Division of Cardiology, Labatt Family Heart Centre, Hospital for Sick Children, Toronto, Ontario, Canada; Division of Cardiovascular Surgery, Labatt Family Heart Centre, Hospital for Sick Children, Toronto, Ontario, Canada; Division of Cardiology, Labatt Family Heart Centre, Hospital for Sick Children, Toronto, Ontario, Canada; Division of Cardiology, Labatt Family Heart Centre, Hospital for Sick Children, Toronto, Ontario, Canada; Department of Pediatric and Adult Congenital Cardiology, Bordeaux University Hospital (CHU), Pessac, France; Division of Cardiology, Labatt Family Heart Centre, Hospital for Sick Children, Toronto, Ontario, Canada; Department of Anesthesia and Pain Medicine, The Hospital for Sick Children, Toronto, Ontario, Canada; Division of Cardiovascular Surgery, Labatt Family Heart Centre, Hospital for Sick Children, Toronto, Ontario, Canada; Division of Cardiology, Labatt Family Heart Centre, Hospital for Sick Children, Toronto, Ontario, Canada; Division of Cardiology, Labatt Family Heart Centre, Hospital for Sick Children, Toronto, Ontario, Canada; Department of Pediatric and Adult Congenital Cardiology, Bordeaux University Hospital (CHU), Pessac, France

**Keywords:** myocardial perfusion, ultrafast power Doppler, histological correlation, right ventricle

## Abstract

**Aims:**

Myocardial vascular remodeling is a key feature in various diseases associated with right ventricular (RV) pressure overload but its non-invasive assessment remains challenging. The new technique attenuation-compensated fractional moving blood volume (acFMBV) based on ultrafast power Doppler (UPD) was developed for myocardial blood volume quantification, linked to RV remodeling and capillary density, but not validated *in vivo*.

**Objectives:**

To quantify RV myocardial blood volume across the cardiac cycle using UPD in rodent models, with histopathological validation, and assess its feasibility in human patients.

**Methods and results:**

Twelve rats (sex ratio 1/1) were randomized to pulmonary artery banding (PAB) (*n* = 6) or sham controls (*n* = 6). After 6 weeks, UPD-derived acFMBV was measured in the RV lateral wall, representing myocardial blood volume. Capillary density was quantified via CD31 immunostaining, and its correlation with mid-diastolic acFMBV was evaluated. UPD was also applied in seven healthy human volunteers and seven age-matched patients with RV pressure overload. Mid-diastolic acFMBV in PAB rats was significantly lower than in controls (5.3% ± 0.9% vs. 7.8% ± 1.2%, *P* < 0.05), correlating with reduced capillary density (*r* = 0.78, *P* < 0.01). In humans, mid-diastolic acFMBV was also lower in RV pressure overload patients compared with healthy volunteers (4.8% ± 0.7% vs. 3.1% ± 2.3%, respectively, *P* = 0.03).

**Conclusion:**

In this pilot study, UPD-based acFMBV correlates with histological capillary density, supporting its potential as a reliable, non-invasive tool for quantifying RV myocardial blood volume in clinical settings. These observations will need to be confirmed in studies with larger sample sizes.

**Condensed abstract:**

Myocardial vascular remodeling in right ventricle (RV) pressure overload is difficult to assess non-invasively. This study validated attenuation-compensated fractional moving blood volume (acFMBV) using ultrafast power Doppler (UPD) to quantify RV myocardial blood volume. Twelve rats underwent pulmonary artery banding (PAB) or sham surgery, with acFMBV measured and correlated with capillary density. PAB rats had lower mid-diastolic acFMBV and capillary density (*r* = 0.78, *P* < 0.01). In humans, RV pressure-overload patients showed reduced acFMBV compared with healthy volunteers (*P* = 0.03). UPD-based acFMBV correlates with histology, supporting its use as a non-invasive tool for assessing RV perfusion.

## Graphical Abstract

**Graphical Abstract:**
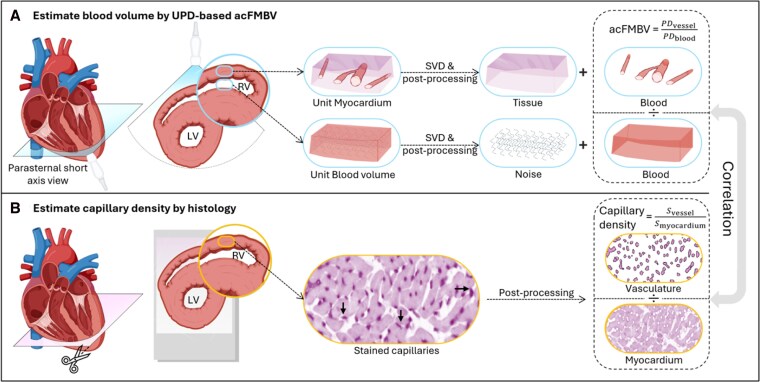
Estimation of blood volume by UPD and histology method. (a) Estimate blood volume by UPD-based acFMBV in RV middle endocardium. Post-processing was applied in ROIs for PD values that contain blood signals. The acFMBV was calculated as the ratio of PD values in ROIs, representing the percentage of blood volume in ROI in RV myocardium. (b) Estimate capillary density by histology, as defined by the ratio of pixel numbers between stained capillaries and the whole myocardium area. acFMBV: attenuation compensated fractional moving blood volume; LV: left ventricle; PD: power Doppler; ROI: region of interest; RV: right ventricle; SVD: singular value decomposition; UPD: ultrafast power Doppler.

## Introduction

Right ventricular (RV) ischemia is associated with RV dysfunction in various heart diseases related to RV pressure overload, such as congenital heart defects and pulmonary arterial hypertension.^1–3^ The outcomes of these patients are determined by RV adaptation mechanisms, which generally progress through stages including initial RV hypertrophy, dilation, followed by systolic and/or diastolic dysfunction.^1,2,4,5^ Adequate maintenance of cardiac output is associated with slower disease progression and improved survival.^6^ However, the mechanisms of RV adaptation remain poorly understood due to the wide heterogeneity in individual responses, rates of progression, and the severity of the RV pressure overload. Moreover, the limited availability of diagnostic tools for quantifying these processes hinders assessments.^1,5,6^ With increasing evidence, myocardial perfusion changes with vasculature remodeling and proliferation (leading to RV oxygen supply/demand mismatch) are one of the key features in hypertrophic responses and RV remodeling.^7,8^ Thus, quantifying myocardial perfusion may better characterize RV remodeling and (mal)adaptation, potentially leading to better risk stratification and management. However, non-invasive quantification of RV myocardial perfusion remains challenging, particularly in newborns and young patients.^9^

An alternative approach to estimating myocardial vascular remodeling is to assess myocardial blood volume as it relates to capillary density. Power Doppler ultrasound has been shown to correlate with the local red blood cell (RBC) concentrations, is angle independent, and highly sensitive to slow blood flow, making it a promising tool for blood volume measurement.^10–12^ It has been applied to identify increased capillary density, for example in cases of inflammation and tumor angiogenesis.^13–15^ However, absolute quantitative measurements with Power Doppler remains challenging due to the attenuation caused by depth, elevation focus, and the presence of multilayers of biological tissue along the ultrasound wave propagation path.^16^

Currently, the only validated quantitative volume measurement based on power Doppler is the fractional moving blood volume (FMBV).^17–19^ This approach standardizes power Doppler measurements by referencing a maximum power Doppler signal obtained from 100% blood (or moving blood) at a similar depth and position, ensuring comparable wave propagation and tissue absorption conditions. By using this ratio-based approach, depth- and tissue-related attenuation effects are canceled, allowing for interobserver comparisons. FMBV provides a percentage of blood volume and has been successfully validated in studies involving the kidney, brain, and placenta, in both animal models and human subjects.^17,18,20^ However, as a natural reference for the maximum Power Doppler signal containing 100% blood, the cardiac chambers do not meet the necessary criteria of being at the same depth as the myocardium and having comparable tissue layers under the transducer. To assess myocardial blood volume using FMBV, Zhang *et al.* developed an optimized method based on ultrafast power Doppler (UPD), termed attenuation-compensated fractional moving blood volume (acFMBV), which enables the selection of a reference maximum blood volume from chambers located at varying depths relative to the myocardium of interest [see *Graphical abstract*, (a)].^16^ While this method has been successfully applied to neonates,^16,21^ it has yet to undergo quantitative validation *in vivo*.

Another major challenge in applying FMBV to the myocardium, compared with other organs, is the intensive motion of the myocardium and the varied blood flow. Previous studies measured FMBV in large visible vessels with stationary or controlled flow. In contrast, the majority of blood volume within the myocardium resides in capillary beds smaller than the resolution of ultrasound imaging (around 200 μm) which are typically not visible in ultrasound cardiac imaging. Additionally, flow velocity and RBC distribution within myocardial capillaries are not temporally uniform throughout the cardiac cycle.^22^

In this study, we validated UPD-based acFMBV for myocardial blood volume quantification by correlating it with myocardial capillary density assessed by immunohistochemistry in rodent models of RV pressure overload. Given the similarity in the densities of blood and myocardium (1.043–1.060 and 1.055 g/mL respectively^23,24^) myocardial capillary density (defined as vasculature to myocardium cross-sectional ratio) also represents a percentage of blood volume in the myocardium [see *Graphical abstract*, (b)]. Consequently, myocardial capillary density is a natural reference standard for acFMBV-based myocardial blood volume measurements. This study aims to demonstrate the feasibility of quantifying blood volume in rapidly moving myocardium and in capillaries. At last, we applied acFMBV to patients with RV perfusion overload, demonstrating the feasibility of quantitative RV blood volume assessment in humans.

## Materials and methods

### Animal protocol

**Figure 1:**
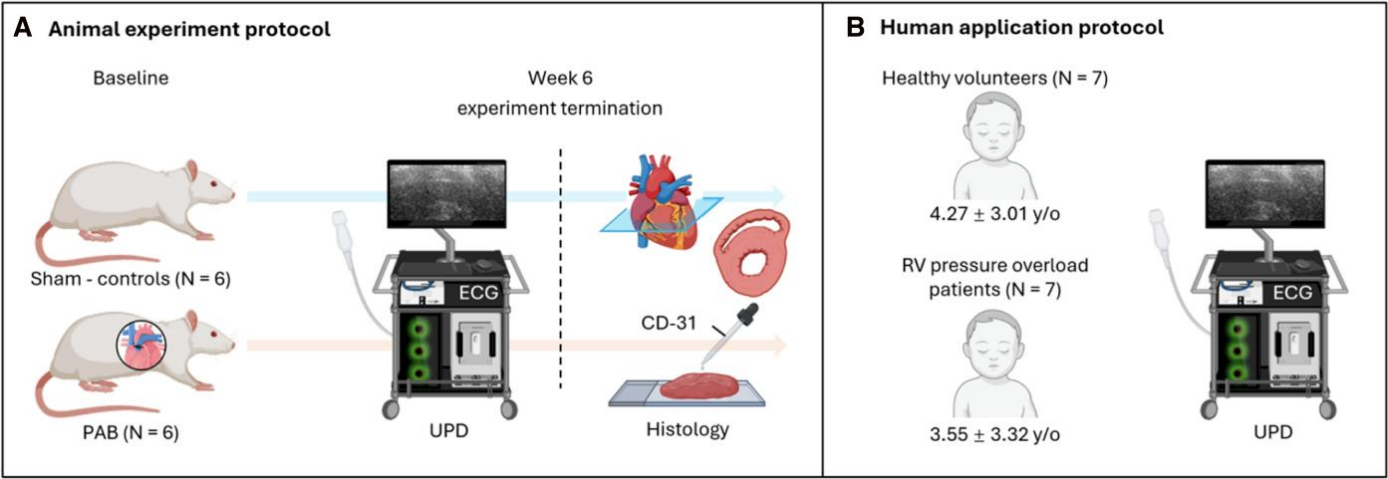
Animal experiment protocol and human application protocol. (*A*) Animal experiment protocol. (*B*) Human application protocol for transthoracic UPD. ECG: electrocardiogram; PAB: pulmonary artery banding; RV: right ventricular; UPD: ultrafast power Doppler.

Twelve (sex ratio 1/1) 6–8-weeks-old Sprague-Dawley rats (Charles River, Senneville, Canada) were randomly assigned to pulmonary artery banding (PAB, *n* = 6), or sham left thoracotomy (control group, *n* = 6). Animals were housed in a controlled environment with a 12-h light–dark cycle, with free access to food and water. At the experiment termination, 6 weeks after surgery, animals were euthanized, and the heart was harvested and processed for histological analysis. Body weight and heart rate were measured at baseline and then weekly until the experiment termination. Body surface area was calculated using Meeh’s formula^25^ (see *Figure 1A*). The animal experiment protocol was approved by the Hospital for Sick Children Research animal ethics committee (Study Approval #59576).

#### PAB model

After anesthesia induction with 4% isoflurane, rats underwent oral intubation and mechanical ventilation. Anesthesia was maintained using 2% isoflurane driven by 100% oxygen (2 L/min). A left anterior thoracotomy in the third intercostal space was performed, the pericardium was opened, and blunt dissection was used to mobilize the main pulmonary artery. A small surgical clip (LT100 ETHICON) was half-closed (inner diameter of 18G) around the main pulmonary artery using a clip applier (LX107 ETHICON) with a stopper to achieve a standardized degree of PA constriction, as previously described.^26–28^ The rats were maintained for 6 weeks. Growth of the animal led to increasing RV pressure overload with animal growth.

#### Sham controls

Sham animals underwent left thoracotomy, pericardiotomy, and pulmonary artery mobilization by blunt dissection under the same anesthetic conditions, without additional surgical steps.

#### Ultrafast power Doppler

**Figure 2:**
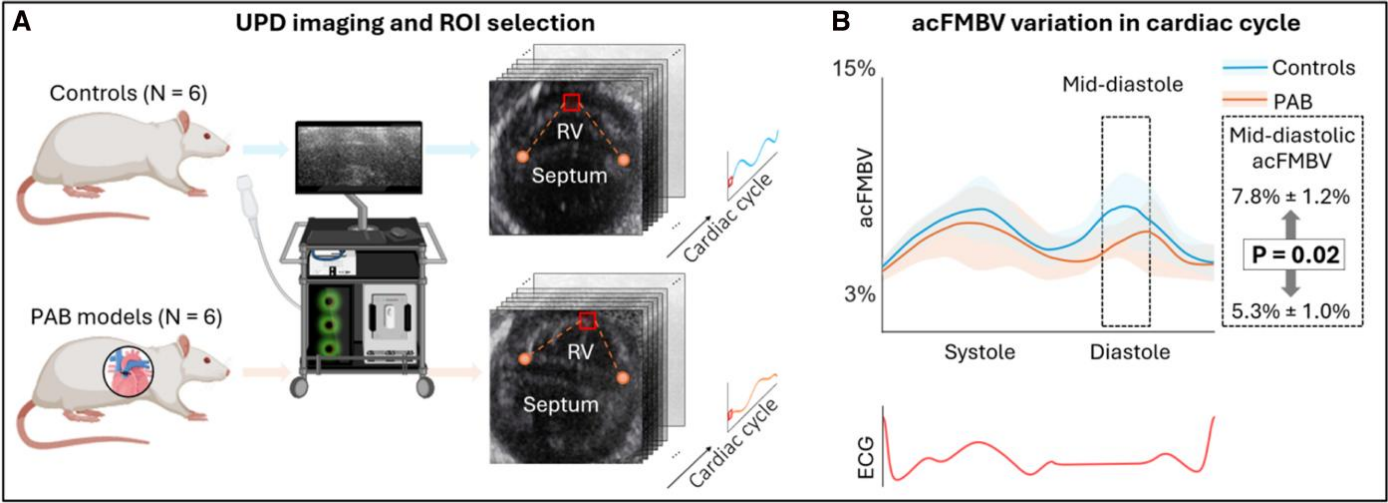
Results of blood volume in RV endocardium accessed by UPD-based acFMBV. (*A*) Ultrafast sequences were acquired from controls and PAB rats. ROIs in red boxes were selected in the middle endocardium of the RV lateral wall according to the intersections of the septum and RV free wall, shown in orange markers. The acFMBV in ROIs was derived throughout the cardiac cycle. (*B*) acFMBV variations in controls and PAB rats. acFMBV: attenuation compensated fractional moving blood volume; ROI: region of interest; RV: right ventricle; UPD: ultrafast power Doppler.

Ultrafast acquisitions were performed using a programmable ultrafast ultrasound scanner (Vantage 256, Verasonics Inc., Kirkland, WA, USA) with an 18.75 MHz linear array probe (L22-14vX, Verasonics Inc.). The ultrafast sequence contained seven compounded tilted plane waves (5°, 3.3°, 1.6°, 0°, −1.6°, −3.3°, −5°) with a 19.6 kHz pulse repetition frequency and therefore 2800 frames per second, using sliding window method.^29,30^ The acquisitions were ECG-triggered automatically by the peak of the R wave and last 530 ms which covered around two cardiac cycles. Three parasternal short axis views centered on RV at the mid-ventricle level (median segment) were acquired for each rat. The first cardiac cycle was extracted between R waves in ECG and equally divided into 9.5 ms (50 frames) sliding ensemble with 40% overlaps.^31^ In each ensemble, a trained analyst manually identified the junctions between the interventricular septum at both ends and the RV free wall based on the temporal-averaged image of 50 frames. A region of interest (ROI) of 60 × 40 pixels was then positioned in the endocardium such that it was equidistant from the two junctions (see *Figure 2A*). The distance of the ROI from the epicardial might be manually slightly adjusted if necessary to avoid inclusion of the blood pool and trabeculations. The acFMBV value was derived as the power Doppler ratio in ROI ($PD_{\mathrm{ROI}}$) and reference region ($PD_{\mathrm{reference}\;}$), and compensated for the depth and elevation focus of power Doppler by an exponential correcting factor *C* (see^16^ for detailed derivation of correcting factor):


$$\{\begin{array}{c} acFMBV=C\times \frac{PD_{\mathrm{ROI}}}{PD_{\mathrm{reference}}}\\ C=\frac{I_{\mathrm{phantom}}(Z_{\mathrm{ROI}})}{I_{\mathrm{phantom}}(Z_{\mathrm{reference}})}e^{-4f_{c}(\alpha_{\mathrm{blood}}-\alpha_{\mathrm{ph}})(z_{\mathrm{ROI}}-z_{\mathrm{REF}})} \end{array},$$

where $I_{\mathrm{phantom}}(Z_{\mathrm{ROI}})$ and $I_{\mathrm{phantom}}(Z_{\mathrm{reference}})$ represented the intensity of the IQ signal averaged at depth of ROI and reference region, respectively ($Z_{\mathrm{ROI}}$, $Z_{\mathrm{reference}}$), acquired with the same transducer and sequence parameters in a homogenous phantom. Meanwhile, $f_{c}$ is the center frequency of the transmitted ultrasound pulse, $\alpha_{\;ph}$ is the effective attenuation coefficient of the phantom given by the phantom manufacturer, and $\alpha_{\mathrm{blood}}$ is the attenuation coefficient of the blood in the ventricle chamber.^32–34^ Each ensemble in the UPD acquisition was post-processed for acFMBV variation throughout the cardiac cycle (see *Figure 2A*).

To assess reproducibility, the eighth and ninth ensembles (during diastole) from each acquisition in control group were reanalyzed anonymously. A new ROI was manually selected with a random size in the mid-endocardium of the RV lateral wall to re-calculate acFMBV. Bland–Altman analysis was performed by pairwise comparison between the original standard-sized ROIs and re-analyzed random-sized ROIs.

#### Histology

**Figure 3:**
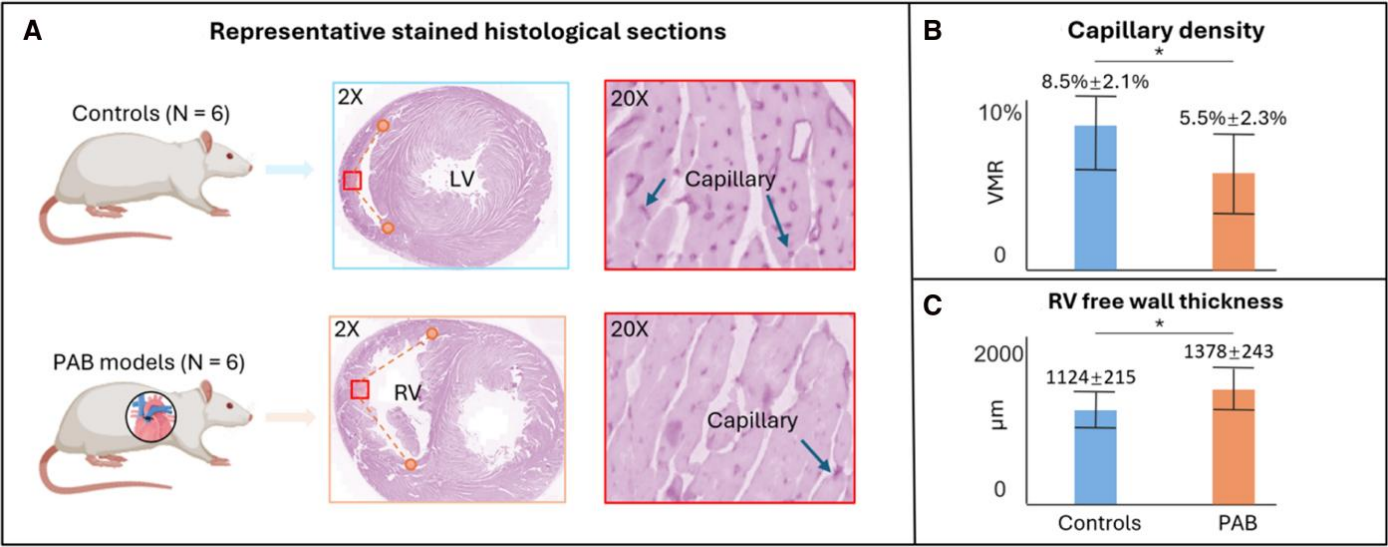
Results of capillary density by histology analysis. (*A*) shows representative stained heart histological sections in controls and PAB rats (at 2× magnification), with ROI in RV mid-endocardium (red boxes, located equidistant from the intersections of the septum and RV free wall, shown as orange lines). At 20× magnification, capillaries were stained in dark pink for further post-processing. (*B*) Capillary density in controls and PAB models. (*C*) RV free wall thickness in controls and PAB models. PAB: pulmonary artery banding; ROI: region of interest; RV: right ventricle; VMR: vessel/myocardium ratio.

Both PAB and control groups underwent deep anesthesia with 4% isoflurane and were euthanized after 6 weeks. Hearts were removed quickly by transecting inferior vena cava, superior vena cava, aorta, and pulmonary artery after sternotomy. Saline solution was then used to flush the coronary arteries and stop the heart during relaxation.^35^ Transverse sections (5 mm) of the hearts were fixed in 10% formalin for 24 h, dehydrated, and embedded in paraffin; 4-μm microtome sections were prepared (Leica Microsystems A/S, Herlev, Denmark). After de-paraffinization, antigen retrieval was performed and then incubated with 0.3% hydrogen peroxide to block endogenous peroxidase activity. The sections were then incubated with 10% BSA for 15 min, followed by incubation with CD31 primary antibodies (Abcam, Cambridge, UK) at 4°C overnight. Subsequently, sections were incubated with a matching biotinylated secondary antibody (Vector, Burlingame, CA) for 45 min at room temperature. Negative controls were performed for all immunological staining by omission of the primary antibody. The sections were viewed and photographed using a Zeiss LSM880 Airyscan microscope and captured by CaseViewer, 3DHISTECH Ltd, Hungary. MATLAB 2022b (The MathWorks Inc., Natick, MA, USA) was used to process the captured images. Capillaries were visualized after CD31 immunostaining and observed clearly under 20 × magnification (see *Figure 3A*). The capillary density, defined as vessel/myocardium cross-section ratio, was calculated and averaged as the ratio of the binary-masked vessel pixel numbers and the total ROI pixel numbers (area).^4,36^ To ensure that the location for capillary density measurement was aligned with the ultrasound ROIs, a region (approximately 1300 µm × 1300 µm, adjusted to account for variations in endocardial thickness across specimens) on the endocardium of the RV lateral wall was selected so that it was equidistant from the junctions of the interventricular septum and the right ventricular free wall, manually identified by the same analyst under 2× magnification (see *Figure 3A*). To avoid large arteries/veins, fibroadipose tissue, or sectional dry cracks, 30 ROIs were then randomly selected in this area under 20× magnification. The histological images were converted to grayscale, and a manually selected threshold (gray level < 130) was applied to generate a binary mask for separating vascular lumina from the surrounding tissue. The capillary density was calculated and averaged over 30 ROIs.

#### Statistical analysis

Two-sample Student’s *t*-tests were used to compare parameters of capillary density, diastolic acFMBV, RV/left ventricle wall thickness, and other animal characteristics between groups. Pearson association test was applied between histological capillary density and diastolic acFMBV for correlation analysis. *P*-values ≤ 0.05 were considered statistically significant. Variables are presented as mean ± SD.

### Human application

#### Patient cohort

We prospectively enrolled seven children (3.55 ± 3.32 y/o) diagnosed with pulmonary valve stenosis with clinical indication for transcatheter valve balloon dilatation (patient group named ‘RV pressure overload patients’). Three parasternal short axis view UPD acquisitions were acquired for each patient for acFMBV post-processing, before the intervention. Seven sex- and age-matched healthy volunteers (4.27 ± 3.01 y/o) with normal cardiac function and unremarkable medical history underwent the same ultrasound protocol and were included for comparison (see *Figure 1B*). This study was approved by The Hospital for Sick Children research ethics board (REB number: 1 000 070 089), and all parents gave written informed consent.

#### Ultrafast power Doppler

The ultrafast acquisition methodology was similar to the animal experiment protocol. A programmable ultrafast ultrasound system (Vantage 256, Verasonics Inc., Kirkland, WA, USA) in conjunction with a 5.7 MHz phased array probe (GE 6S-D, GE Healthcare, USA). Seven diverging waves were used for coherently compounding and the five virtual sources of diverging waves were positioned as in Correia *et al.*^29,37^ The acquisitions lasted 842 ms at 1996-Hz framerate, so that at least one cardiac cycle was recorded. At least three median segment RV-centered parasternal short axis acquisitions were obtained for each patient, triggered by the R wave while ECG was recorded. The ROI selection, cluttering filtering, acFMBV computation, and reproducibility were the same as the animal experiment protocol. The reproducibility was tested in the first two ensembles in diastole (defined by the myocardium deformation on the B-Mode cine loop manually).

#### Statistical analysis

Student’s *t*-tests were used to compare diastolic acFMBV (mean ± SD) between groups. The statistical analysis on acFMBV between healthy volunteers and RV pressure overload patients was applied during mid-diastole, same as the animal experiment, for the comparison. *P*-values ≤ 0.05 were considered statistically significant. All image post-processing and statistical analysis were performed using MATLAB 2022b (The MathWorks Inc., Natick, MA, USA).

## Results

### Animal experiment results

All animals underwent the full experimental protocol for acFMBV measurement and histological analysis. At the 6-week observation time point, there were no significant differences between PAB rats and controls in terms of body weight (440 ± 133 g vs. 391 ± 56 g, *P* = 0.41) and body surface area (525 ± 100 cm^2^ vs. 524 ± 49 cm^2^, *P* = 0.77). The heart rates of PAB rats and controls were similar (323 ± 6 vs. 327 ± 26 beats per minute, *P* = 0.71).

#### Ultrafast power Doppler

The averaged acFMBV variations in cardiac cycle for PAB rats and controls were shown in *Figure 2B*. In controls, the acFMBV changes showed a bimodal pattern, with the peak in systole similar to the peak in diastole (systole peak: 8.60% ± 1.43%; diastole peak: 8.60% ± 1.87%; *P* = 0.99). In the PAB group, the systolic peak value was slightly higher than the diastolic peak value (systole peak: 8.14% ± 1.84%; diastole peak: 7.40% ± 1.79%; *P* = 0.48), resulting in a bimodal pattern but less variation between systole and diastole (variation is defined as the extent to which both peaks exceed the mean value, calculated as [systolic peak—average] + [diastolic peak—average]) (4.96% vs. 5.18%) During mid-diastole, the mean mid-diastolic acFMBV of PAB rats was 5.89% ± 2.08% lower than controls was 7.89% ± 1.82%, *P* = 0.02 (*Figure 2B* and *Table 1*). For reproducibility, Bland–Altman analysis demonstrated the agreement between the two measurements, with a 95% confidence interval of [−3.8%, 4.9%] and a mean difference of 0.6% (*Figure 6A*).

**Table 1:** Animal experiment results—acFMBV

	Controls (*n* = 6)	PAB-6wk (*n* = 6)	*P*-value
Systolic peak	8.60%±1.43%	8.14%±1.84%	0.348
Diastolic peak	8.60%±1.87%	7.40%±1.79%	0.171
Mid-diastolic mean	7.81%±1.24%	5.32%±0.88%	0.024

Values are mean ± STD.

acFMBV: attenuation compensated fractional moving blood volume; PAB: pulmonary artery banding.

#### Histological analysis

The heart geometry altered markedly after PAB. The interventricular septum flattened, and RV free-wall thickness increased^28,38^ (see *Figure 3A*). Histological analysis confirmed significantly increased RV free wall thickness in PAB rats compared with controls (PAB: 1378 ± 243 µm; controls: 1124 ± 215 µm; *P* = 0.01), whereas left ventricle free wall thickness was similar between groups (PAB: 2710 ± 270 µm; controls: 2759 ± 336 µm; *P* = 0.336), see *Figure 3B*.

The mean capillary density in the median segment of the RV lateral wall was 5.5% ± 2.3% vs. 8.5% ± 2.1% in PAB rats vs. controls, respectively (*P* = 0.001), see *Figure 3B*.

#### Correlation

**Figure 4:**
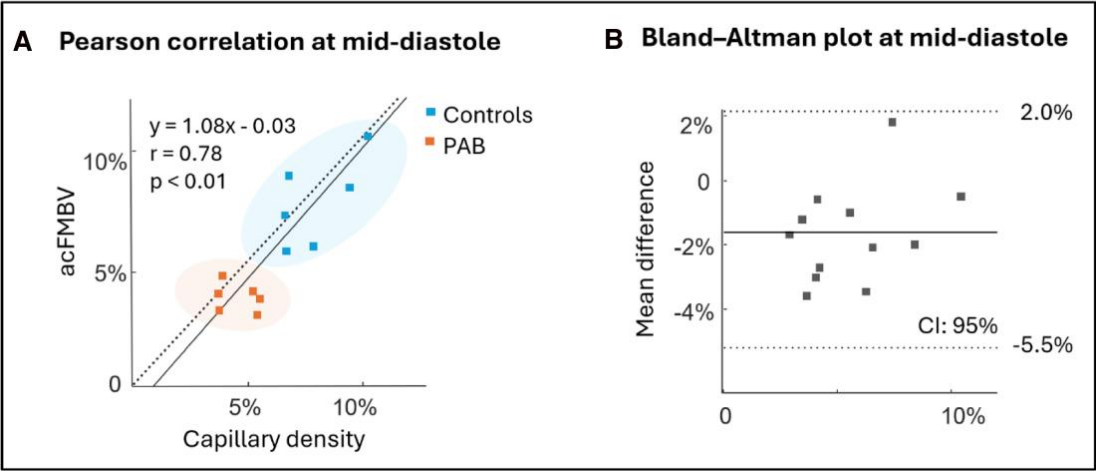
Correlation between RV myocardium blood volume estimation from UPD-based acFMBV and histology methods. (*A*) Pearson correlation scatter between diastolic acFMBV and capillary density. (*B*) Bland–Altman analysis between acFMBV and capillary density, all measurements are within the 95% confidence interval. acFMBV: attenuation compensated fractional moving blood volume.

There was a significant positive correlation between mid-diastolic acFMBV and histological capillary density (*r* = 0.78, *P* < 0.01), (see *Figure 4A*). Meanwhile, Bland–Altman analysis showed a mean pair difference of −1.78% between the two methods, all measurements are within the 95% confidence interval [(−5.5%, 2.0%)] (see *Figure 4B*).

### Human results

**Figure 5:**
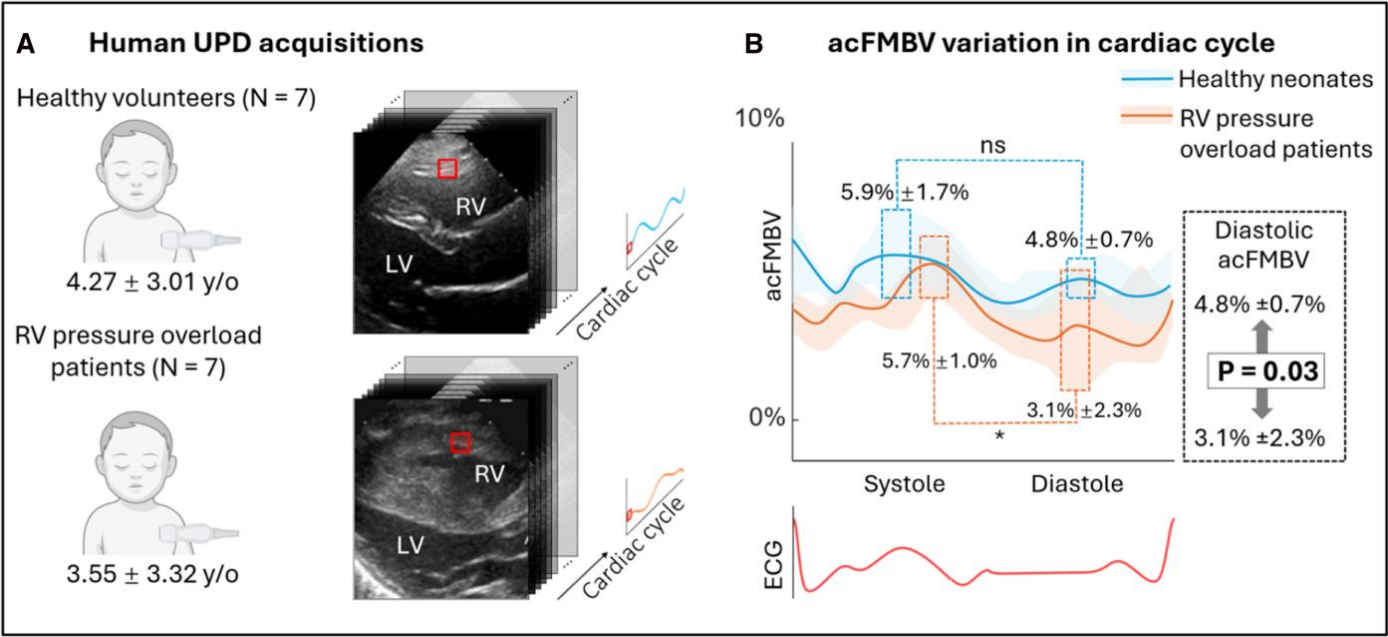
Results of capillary density by histology analysis. (*A*) Ultrafast ultrasound images acquired from healthy volunteers and RV pressure overload patients. ROI shown in red boxes was selected in the middle of RV endocardium. (*B*) The acFMBV was computed throughout the cardiac cycle with a bimodal pattern. acFMBV: attenuation compensated fractional moving blood volume; LV: left ventricle; ROI: region of interest; RV: right ventricle; UPD: ultrafast power Doppler.

**Figure 6:**
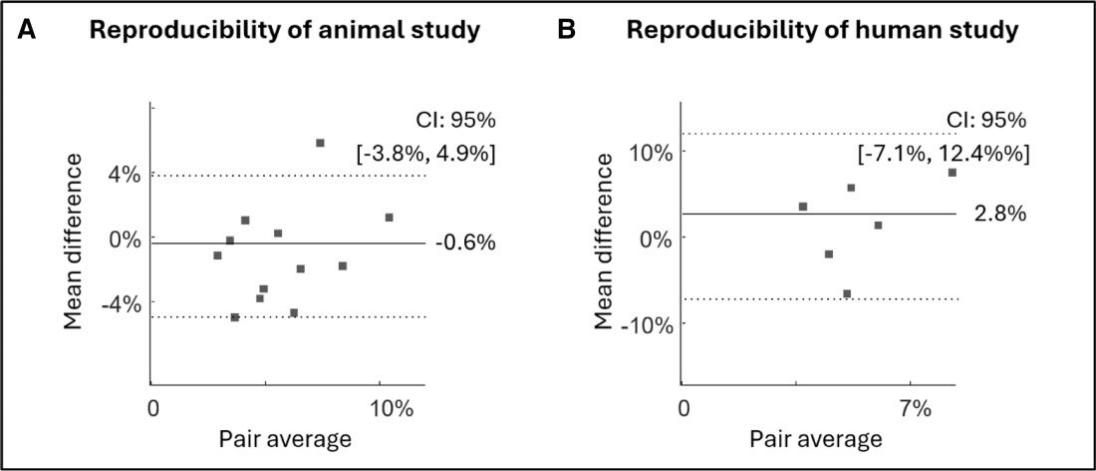
Reproducibility of acFMBV measurement in animal and human studies. (*A*) Bland–Altman analysis of animal data in mid-diastole. (*B*) Bland–Altman analysis of human data in mid-diastole.

Seven healthy volunteers (mean age: 2.66 ± 3.09 years) and seven RV pressure overload patients (mean age: 5.34 ± 5.80 years, *P* = 0.75 vs. healthy controls) were included. Echocardiographic and hemodynamical parameters are presented in *Table 2*. The human acFMBV variation patterns were similar to the patterns in animal experiments. In healthy volunteers, the acFMBV pattern followed a bimodal pattern with a peak in mid-systole (5.9% ± 1.7%) and a similar peak in mid-diastole (4.8% ± 0.7%) (*P* = 0.474). In RV pressure overload patients, the acFMBV pattern was blurred with a significant higher peak in systole compared with diastole (systole: 5.7% ± 1.0% vs. diastole: 3.1% ± 2.3%; *P* = 0.041), see *Figure 5*. During mid-diastole, the average acFMBV of healthy volunteers was significantly higher than the average acFMBV of RV pressure overload patients (4.8% ± 0.7% vs. 3.1% ± 2.3%, respectively, *P* = 0.03). For reproducibility, Bland–Altman analysis demonstrated the agreement between the two measurements, with a 95% confidence interval of [−7.1%, 12.4%] and a mean difference of 2.8% (*Figure 6B*). No statistically significant correlation was found between acFMBV data and echocardiography or hemodynamic data (all *P* > 0.05).

**Table 2:** Children protocol—echocardiography and hemodynamic data

	RVPO (*n* = 7)	HV (*n* = 7)	*P* value
**Echocardiography**			
RV FWT, mm	3.1 ± 1.2	2.3 ± 0.5	0.01
RV EDD, mm	21.7 ± 9.4	14.2 ± 3.8	0.01
RV EDD, z-score	3.2 ± 2.0	0.7 ± 0.8	<0.01
RV FAC, %	40 ± 6	39 ± 6	0.37
RV TAPSE, cm	1.36 ± 0.37	1.99 ± 0.26	<0.01
RV E/E’	6.5 ± 1.9	3.8 ± 0.8	<0.01
RVSP, mm Hg	60 ± 18	19 ± 6	<0.01
LVEF, %	65 ± 6	64 ± 3	0.78
**Hemodynamics**			
RVSP (mmHg)	57 ± 25	—	
RV dP/dt_max_ (mmHg/s)	531 ± 144	—	
RV Ees (mmHg/mL)	2.73 ± 2.18	—	
RV EDP (mmHg)	11 ± 4	—	
RV dP/dt_min_ (mmHg/s)	−501 ± 99	—	
RV tau (s)	45.1 ± 8.1	—	
Cardiac index (L/min/m^2^)	3.80 ± 0.41	—	

Values are mean ± SD.

EDD: end-diastolic diameter; FAC: fractional area change; HV: healthy volunteers; LV: left ventricle; LVEF: left ventricular ejection fraction; NWV: natural wave velocity; PAB: pulmonary artery banding; PVC: pulmonary valve closure; RV: right ventricle; RV FAC: RV fractional area change; RV FWT: RV free wall thickness; RVPO: right ventricular pressure overload; TAPSE: tricuspid annular plane systolic excursion.

## Discussion

In this study, quantitative myocardial blood volume assessed by acFMBV is demonstrated to correlate with the histological reference of vessel cross-sectional area. Our results show that in the animal experiments, both acFMBV and capillary density decreased in PAB rats compared with controls. Further, mid-diastolic acFMBV correlated with histological capillary density (*r* = 0.78, *P* < 0.01). Similarly, in humans, acFMBV in healthy individuals was significantly higher than in patients with RV pressure overload. The acFMBV variation patterns throughout the cardiac cycle in animal models and in humans were highly consistent.

### Patterns of acFMBV variation in UPD

In our study, the patterns of RV acFMBV variation in healthy animal models and human volunteers were consistent with results in our previous FMBV studies and the normal RV perfusion rates, showing bimodal patterns, with one peak in systole and another in diastole.^21,39^ Similar bimodal patterns of normal heart perfusion (mL/g/min) and volume (mL/min) variation have also been observed in studies using flow probes, positron emission tomography, and contrast-free magnetic resonance imaging, in dogs and in human studies.^40–42^ However, in PAB rats and RV pressure overload patients, this bimodal pattern changed with loss of the acFMBV variation and reduced diastolic blood volume. The acFMBV variation trended to flatten between the end of systole and the early diastole.

UPD is proportional to the local number of RBCs and does not provide velocity information. Therefore, UPD-based acFMBV represents blood volume but not perfusion or flow rate. Given the uniform size of the ROI chosen throughout the cardiac cycle and the standardization of calculations, the variation in acFMBV reflect changes in the number of RBCs in a unit volume of myocardium. This variation may result from several factors:

1. the arteriolar and periarteriolar vessels below our sensitivity included in the ROI are influenced by myocardial contraction (tissue pressure).^39^ Due to higher surrounding interstitial pressure, endomyocardial vasculature may be more significantly deformed by pressure than epicardial vasculature.^43^ However, the acFMBV blood volume in diastole is not substantially higher than in systole, as similar peaks are observed during both phases of the cardiac cycle. Additionally, since most blood volume resides in the capillaries, the small amount of arteriolar blood included in the ROI is not a major factor in acFMBV fluctuations;
2. increasing capillary density as the distances between capillaries decrease, driven by the reduction in cardiomyocyte diameter during the cardiac cycle. However, since myocardial deformation does not follow a bimodal pattern, this is not a primary cause of acFMBV variation;
3. heterogeneity in capillary perfusion and regional adjustments influence RBC concentration, density distribution, and velocity.^22,44–46^ The variation in RBC flow distribution has been demonstrated to result from the bifurcation points of the microvascular network.^47^ The diameter and angle of each bifurcation determine the proportion of plasma and RBCs that enters each branch.^48,49^ Experiments found various intracapillary spacing between RBCs, averaging over 5 µm at rest.^46,50^ During reactive hyperemia, coronary capillaries undergo significant dilation, leading to increased blood flow and higher RBC line density.^51^ Capillary endothelial cell glycocalyx, endothelial cell vasodilators, and pericytes regulate the distribution of RBCs and thus perfusion, independently of arteriolar.^22^ The fluctuations in acFMBV we observed may reflect these complex autoregulatory mechanisms.

The regulation of myocardial perfusion is highly complex and involves multiple factors, including extravascular compression (tissue pressure), the coronary perfusion gradient, myogenic mechanisms, local metabolic demands, endothelial function, and hormonal influences.^39,52–54^ However, only a few of these factors are directly related to changes in blood volume. These hypotheses need to be explored through further studies.

### Histological results and capillary density

A plausible mechanism for the reduced acFMBV was the observed capillary rarefaction as quantified by capillary density.^4^ As shown in *Figure 3*, there was a significant vessel density drop in PAB rats with a lower capillary density compared with controls. This phenomenon has been described as a transition from adaptive hypertrophy to maladaptive failure during chronic and progressive pressure overload situations.^55–57^ Therefore, non-invasive quantification of myocardial blood volume at the bedside using acFMBV may therefore be useful in detecting development of RV failure.

### Correlation between mid-diastolic acFMBV and capillary density

Pearson correlation and Bland–Altman analysis showed a significant positive correlation and consistency between acFMBV and histologic methods. As described above, acFMBV is the ratio of myocardial blood volume over the unit volume of myocardium (if blood was perfectly extracted and compensated from the myocardium).^16^ Here, the volume could be approximated as a two-dimensional area (or cross-sectional area) by assuming negligible elevational thickness of the imaging plane.^58^ Therefore, histological capillary density can be considered as a reference technique for acFMBV validation.^59^

During euthanasia, 4% isoflurane was used for deep anesthesia, which can cause decreased heart rate and blood pressure in rats.^60^ Likewise, as a potent coronary vasodilator,^61^ high concentrations of isoflurane can induce negative inotropic and chronotropic effects by affecting ion channels and intracellular signaling pathways.^62,63^ After exposure to isoflurane, incubation in saline gradually slowed the heart rate, relaxed the heart muscle, and finally stopped the heart in a relaxed state at mid-diastole.^35^ Therefore, acFMBV and histological capillary density correlated the strongest during mid-diastole and maximal vasodilation as they both were quantified myocardial blood volume/capillarization while the myocardium maximally relaxed.

Alongside the strong correlation, the mean difference between acFMBV and capillary density was slightly negative (−1.78%), see in *Figure 4B*. Honig *et al*. showed that in the normal rat hearts, around 20% of capillaries are not perfused. This may reflect a functional reserve mechanism, conferring the ability to augment flow when needed, for example during hypertrophy or increased diseases.^64–66^ These unperfused capillaries might explain why the mean difference between acFMBV and capillary density was negative, as they did not contribute any blood signal in UPD due to lack of sensitivity.

### Human application

The human RV myocardial blood volume variation patterns align with our previous acFMBV study and previous coronary perfusion studies.^21,39–42^ In healthy individuals, we observed a bimodal pattern with two similar peaks in both systole and diastole. We did not show a higher peak in systole in RV, which might be related to the low signal-to-noise ratio or the limited number of subjects. In patients with RV pressure overload, the acFMBV variation was more likely to be a single peak pattern as the acFMBV in systole was significantly higher than the acFMBV in diastole. The reason might be the dysfunction of capillary autoregulation and the high continuous extravascular compression in the hypertrophy response.^67^

We also found significant differences in diastolic acFMBV between healthy volunteers and RV pressure overload patients. This difference was consistent with the results from animal experiments and may further reflect the physiological changes in RV pressure overload.

The human component of this study was designed as a proof-of-concept investigation to assess feasibility and detect group-level differences in myocardial blood volume using acFMBV. Although correlations between acFMBV and hemodynamic markers of disease severity would be of high clinical interest, the limited sample size and clinical heterogeneity of the pediatric RV pressure overload cohort preclude statistically robust correlation analyses. Larger prospective studies will be required to explore the relationship between myocardial blood volume, hemodynamic severity, and clinical outcomes.

### Study limitations

Our study presents some clinical and technical limitations.

First of all, our observations and statistical analyses are based on limited numbers of subjects in both the animal model (*n* = 6 + 6) and patients (*n* = 7 + 7). These observations will need to be confirmed in studies with larger sample sizes.

Secondly, the compensation technique requires a clear view of the endocardium for ROI selection so that experienced operators are necessary to acquire acFMBV data.

Isoflurane anesthesia, used in the animal experiments, is known to induce coronary vasodilation and alter myocardial hemodynamics; however, all rats were studied under identical anesthetic conditions, preserving the validity of within-species comparisons, while differences in anesthesia status may partly contribute to absolute acFMBV differences between animal and human data.

An additional limitation concerns the physiological interpretation of the systolic acFMBV peak. Although systolic myocardial contraction is known to impede coronary flow due to extravascular compression, acFMBV reflects myocardial blood volume rather than flow velocity. Therefore, a systolic increase in acFMBV does not necessarily imply increased coronary perfusion. The observed systolic peak may result from transient pooling of blood within compliant microvascular compartments under tissue compression, increased apparent capillary density due to myocardial wall thickening, or partial through-plane motion despite careful ROI placement. While several methodological steps were taken to minimize motion-related artifacts, including ensemble-based normalization and consistent mid-endocardial ROI positioning, these effects cannot be entirely excluded. Importantly, the primary quantitative analyses and histological validation were performed during mid-diastole, when myocardial motion and extravascular compression are minimal, supporting the robustness of the main findings. Future studies combining acFMBV with motion tracking or three-dimensional imaging may further clarify the contribution of myocardial deformation and through-plane motion to systolic blood volume measurements.

Technically, acFMBV calculation requires high imaging quality (signal-to-noise ratio) and adequate imaging windows. As discussed previously in the correlation section, blood was assumed to be perfectly extracted from the myocardium and well compensated for power Doppler against depth. However, many factors including heart structural differences, blood cell concentration differences, trabeculations, and rouleaux phenomena could have an impact on absolute quantification. For example, we assumed that the blood attenuation coefficient of each patient was the same at 0.2860 dB·cm^−1^·MHz^−1^ [hemoglobin concentration 15 g dL^−1^, 37°C (5–10) MHz] according to Treeby *et al.* because the hemoglobin concentration was not monitored in healthy volunteers as RV pressure overload patients did during their intervention.^68^ Likely, the blood backscattered coefficient in rats within a frequency range of [4–9] MHz was neither measured due to limited experimental equipment nor directly provided in accessible online resources. We therefore assumed that it fell in a similar range as human blood. However, this may be a potential reason for differences in acFMBV between animals and humans.

Another technical limitation concerns the alignment of the ROIs. The alignment of the ROIs was performed under the assumption that the ultrasound images and the histological data were acquired in the same imaging plane. In practice, the parasternal short-axis views and the histological sections are unlikely to be perfectly coincident, which may introduce small spatial discrepancies.

Regarding reproducibility analysis, the relatively wide limits of agreement observed in the human study reflect the combined influence of multiple factors that affect acFMBV measurements. To minimize spatial variability, we selected relatively large ROIs for averaging. This approach, however, constrained the flexibility of ROI placement during the reproducibility experiment because the new ROI had to avoid overlap with the original one. Given the limited area in pixels in the mid-RV free wall near the apex of the diverging-wave, the new ROIs could shift away from the lateral center and mid-RV free wall, thereby leading the acFMBV variations. In addition, the use of a 5.7 MHz phased-array probe for human myocardial imaging resulted in relatively lower image quality, further affecting acFMBV estimation. These factors represent a primary limitation of the technique when applied in clinic. Future clinical use would require standardized acquisition protocols.

## Conclusion

This study showed a correlation between acFMBV measurement by UPD and histological capillary density in rodent experiments, suggesting that the myocardial blood volume measurement by acFMBV is a reliable quantitative technique. The results of the human study consistent with the animal results demonstrated the feasibility of human application in newborns and children. UPD-based quantitative myocardial blood volume assessment could improve our understanding of adaptive vs. maladaptive RV hypertrophy, offering new perspectives for patient monitoring and treatment. Larger patient recruitment and additional analyzed myocardial segments are needed for further studies.

## Data Availability

The data supporting the findings of this study are available from the corresponding author, Dr. Olivier Villemain, upon reasonable request. He can be contacted at: olovillemain@gmail.com.

## Lead author biography

Naiyuan Zhang (PhD) likes knowledge integration and innovation. He holds a master of applied science in Electrical & Computer Engineering. He also holds a bachelor’s degree in Optoelectronic Information Science and Engineering. He did his PhD in the Villemain Lab (SickKids Research Institute, Toronto, Canada) in the field of ultrasound imaging and medical image processing.
